# Comparison of Treatment Outcomes between Breast Conserving Surgery Followed by Radiotherapy and Mastectomy Alone in Patients with T1-2 Stage and 1-3 Axillary Lymph Nodes in the Era of Modern Adjuvant Systemic Treatments

**DOI:** 10.1371/journal.pone.0163748

**Published:** 2016-09-29

**Authors:** Sang-Won Kim, Mison Chun, Sehwan Han, Yong Sik Jung, Jin Hyuk Choi, Seok Yun Kang, Hyunsoo Jang, Sunmi Jo

**Affiliations:** 1 Department of Radiation Oncology, Ajou University School of Medicine, Suwon, Republic of Korea; 2 Department of Surgery, Ajou University School of Medicine, Suwon, Republic of Korea; 3 Department of Hematology-Oncology, Ajou University School of Medicine, Suwon, Republic of Korea; 4 Department of Radiation Oncology, Dongguk University Gyeongju Hospital, Gyeongju, Republic of Korea; 5 Department of Radiation Oncology, Haeundae Paik Hospital, Inje University School of Medicine, Busan, Republic of Korea; University of North Carolina at Chapel Hill School of Medicine, UNITED STATES

## Abstract

**Purpose:**

The role of postmastectomy radiotherapy in the treatment of T1–2 primary tumor with 1–3 positive lymph nodes is controversial. We compared treatment outcomes between breast conserving surgery followed by radiotherapy (BCS+RT) and total mastectomy alone (TM) in the setting of modern adjuvant systemic treatments.

**Methods:**

Patients with T1–2 primary breast cancer and 1–3 positive lymph nodes who were treated between 2001 and 2011 were divided into 2 groups based on the treatment approach: BCS+RT (*n* = 169) and TM (*n* = 117). All patients received adjuvant chemotherapy including taxanes. Adjuvant endocrine therapy was administered to patients with positive hormone receptors according to their menstrual status.

**Results:**

During a median follow-up of 76.5 months, 21 patients (7.3%) experienced locoregional recurrence as the first event, including 7 patients (4.1%) in the BCS+RT group and 14 patients (12.0%) in the TM group. The 5-year cumulative incidence rate of locoregional recurrence was 2.5% for BCS+RT versus 9.5% for TM (*p* = 0.016). Competing risk regression analysis revealed that TM was associated with a relative risk for locoregional recurrence of 5.347 (*p* = 0.003). TM was also associated with a significantly lower 5-year disease-free survival rate compared with BCS+RT (hazard ratio, 2.024; 95% confidence interval, 1.090–3.759; *p* = 0.026).

**Conclusion:**

To improve treatment outcomes for TM even after modern systemic treatments, postmastectomy radiotherapy might be required for patients with T1–2 primary breast cancer and 1–3 positive lymph nodes.

## Introduction

For early-stage breast cancer, breast-conserving surgery followed by radiotherapy (BCS+RT) and total mastectomy alone (TM) produced similar survival rates in two prospective randomized trials with long-term follow-up [1, 2]. As BCS+RT had an additional cosmetic advantage, these studies resulted in a paradigm shift from TM to BCS+RT for treating early-stage breast cancer. However, a considerable proportion of patients with early-stage breast cancer still undergo mastectomy because of multifocal or multicentric tumors, diffusely scattered microcalcifications, persistent positive margin after repeated attempts at BCS, or patient preference.

The similar treatment outcomes between BCS+RT and TM in early prospective randomized trials suggested that postmastectomy radiotherapy (PMRT) could be omitted as a treatment option for early-stage breast cancer. However, in contrast to node-negative early-stage breast cancer, for which most clinicians agree on the negligible benefit of PMRT, the use of PMRT has been controversial for patients with T1–2 primary breast cancer and 1–3 positive lymph nodes (T1–2/N1). Although early guidelines did not recommend PMRT for the treatment of T1–2/N1 breast cancer because of insufficient evidence [3–6], subsequently published retrospective studies demonstrated that local control and survival could be improved by PMRT in patients with certain high-risk factors [7–14]. The recently updated Oxford overview recommends strong consideration of the routine use of PMRT for patients with T1–2/N1 breast cancer [15–18]. However, this meta-analysis included prospective randomized trials initiated prior to 2000 when antiquated systemic treatments were used, and consequently, its findings do not fit current clinical practice. It is well established that advances in systemic regimens over the last decade have substantially reduced the risk of recurrence in early-stage breast cancer, which has limited the role of adjuvant local treatment.

In this situation, a direct comparison of treatment outcomes between PMRT and TM in the setting of modern adjuvant systemic treatments is needed. However, no prospective randomized trials have been reported. The existing retrospective studies might share biases because the decision to use PMRT was not randomized but instead usually depended upon pathologic characteristics.

Alternatively, indirect comparisons of treatment outcomes between BCS+RT and TM can offer valuable information. Because radiation has been indicated for all patients treated with BCS and PMRT has not been performed in patients with T1–2/N1 breast cancer at out institution, fewer disease-related biases would affect the patterns of radiation use. The results of several studies comparing treatment outcomes between BCS+RT and TM have been previously published, although they analyzed patients before the introduction of taxanes, aromatase inhibitors, and trastuzumab, which improved treatment outcomes in the adjuvant setting [19–21].

The purpose of this study was to compare clinical outcomes of patients with T1–2/N1 breast cancer who were treated with BCS+RT or TM in the era of modern adjuvant systemic treatments.

## Methods

This study was approved by the Institutional Review Board of Ajou University School of Medicine without a requirement for informed consent. We retrospectively reviewed the outcomes of patients with T1–2/N1 breast cancer who underwent curative surgery at our institution between 2001 and 2011. The exclusion criteria were as follows: preoperative chemotherapy, bilateral invasive breast cancer, a past history of malignancy except papillary thyroid cancer, and an absence of follow-up data.

In total, 286 eligible patients were identified. Sentinel lymph node biopsy was performed unless axillary lymph node metastasis was confirmed by fine-needle aspiration biopsy before surgery. Patients with tumor-positive sentinel nodes underwent axillary dissection excluding 11 patients who did not have a positive frozen section and elected not to undergo axillary dissection. These patients were included in the analysis because recently published, large, prospective, randomized trials demonstrated that treatment outcomes did not differ among patients with breast cancer with micrometastatic sentinel lymph nodes based on the decision to undergo axillary dissection [22, 23].

All patients received 6–8 cycles of adjuvant chemotherapy including taxanes. Adjuvant trastuzumab was approved by the Korean Ministry of Food and Drug Safety in June 2007, and it was subsequently administered to 21 patients with human epidermal growth factor receptor 2 (HER2)-positive breast cancer. Adjuvant endocrine therapy was administered to patients with hormone receptor-positive breast cancer according to their menstrual status. A selective estrogen receptor modulator was administered to 166 premenopausal women for 5 years, and an aromatase inhibitor was administered to 64 postmenopausal women for 5 years.

Ipsilateral whole breast irradiation was delivered using tangential beams, with a median total dose of 45 Gy in 25 fractions. An electron beam boost was delivered to the tumor bed plus a margin of 2 cm, with a median dose of 14 Gy in 7 fractions. Elective irradiation to the supraclavicular nodal area was performed in patients with multiple lymph node metastases, lymphovascular space invasion, less than 10 dissected lymph nodes, or a lymph node ratio (the number of tumor-positive lymph nodes divided by the number of dissected lymph nodes) greater than 0.2. For patients who did not undergo axillary lymph node dissection, radiation was delivered to the entire axillary lymph node area. Internal mammary nodal irradiation was not performed.

We divided the patients into 2 groups based on the treatment approach: BCS+RT (*n* = 169) and TM (*n* = 117). The clinicopathological characteristics of the groups are summarized in Table 1. T2 stage, HER2 overexpression, and multiple positive lymph nodes were more common in the TM group (all *p* < 0.05). By contrast, a close resection margin was more common in the BCS+RT group. Other factors were not significantly different between the groups.

**Table 1:** Patient characteristics.

		BCS + RT (%)	Mastectomy (%)	*p* value
Age		median, 46	median, 47	0.560
	<40 year	34 (20.1)	27 (23.1)	
	≥40 year	135 (79.9)	90 (76.9)	
Menstrual status				0.227
	premenopausal	127 (75.1)	80 (68.4)	
	postmenopausal	42 (24.9)	73 (31.6)	
T stage				0.030
	T1	95 (56.2)	50 (42.7)	
	T2	74 (43.8)	67 (57.3)	
Resection margin				<0.001
	≥2 mm	140 (82.8)	113 (96.6)	
	<2 mm	29 (17.2)	4 (3.4)	
LVSI				0.817
	negative	61 (36.1)	42 (35.9)	
	positive	70 (41.4)	52 (44.4)	
	unknown	38 (22.5)	23 (19.7)	
Nuclear grade				0.808
	1	76 (45.0)	49 (41.9)	
	2	77 (45.6)	54 (46.2)	
	3	5 (3.0)	3 (2.6)	
	unknown	11 (6.5)	11 (9.4)	
Histologic grade				0.943
	1	20 (11.8)	15 (12.8)	
	2	67 (39.6)	46 (39.3)	
	3	78 (46.2)	52 (44.4)	
	unknown	4 (2.4)	4 (3.4)	
Estrogen receptor				0.162
	negative	36 (21.3)	34 (29.1)	
	positive	133 (78.7)	83 (70.9)	
Progesterone receptor				0.107
	negative	40 (23.7)	38 (48.7)	
	positive	129 (76.3)	79 (67.5)	
Adjuvant hormone Tx				0.395
	No	28 (16.6)	26 (22.2)	
	SERM	100 (59.2)	68 (58.1)	
	AI	41 (24.3)		
HER2 expression				0.001
	negative	141 (83.4)	77 (65.8)	
	positive	28 (16.6)	40 (34.2)	
Adjuvant trastuzumab*				0.794
	No	20 (71.4)	27 (67.5)	
	Yes	8 (28.6)	13 (32.5)	
Removed LN		median, 17	median, 18	0.383
	≥10	144 (85.2)	104 (88.9)	
	<10	25 (14.8)	13 (11.1)	
Positive LN				0.023
	1	101 (59.8)	51 (43.6)	
	2	39 (23.1)	41 (35.0)	
	3	29 (17.2)	25 (21.4)	
LNR		median, 0.08	median, 0.09	1.000
	≤0.2	145 (85.8)	100 (85.5)	
	>0.2	24 (14.2)	17 (14.5)	
Adjuvant chemotherapy				0.691
	(F)AC #4 → T #4	166 (98.2)	114 (97.4)	
	Others (TAC, AT, TC) #6	3 (1.8)	3 (2.6)	

BCS+RT, breast-conserving surgery followed by radiotherapy; TM, total mastectomy; LVSI, lymphovascular space invasion; Tx, therapy; SERM, selective estrogen receptor modulator; AI, aromatase inhibitor; HER2, human epidermal growth factor receptor 2; LN, lymph node; LNR, lymph node ratio; FAC, fluorouracil, adriamycin, cyclophosphamide; T, taxane; TAC, taxane, adriamycin, cyclophosphamide; AT, adriamycin, taxane; TC, taxane, cyclophosphamide

* Among HER2-positive patients

### Statistical analysis

The Fisher's exact or the chi-square test was used to compare patient characteristics between the treatment groups. Locoregional recurrence (LRR) was defined as the first tumor recurrence in the ipsilateral breast or chest wall, axillary lymph node, internal mammary lymph node, and/or infra-/supraclavicular lymph node area. The LRR rate was estimated using cumulative incidence analysis as described by Gray [24], and the competing risks were distant metastasis, contralateral breast cancer, or death from any cause. A competing risk regression model was used to identify risk factors for LRR [25]. The covariates included in the regression model were age (<40 years vs. ≥40 years), T stage, resection margin, nuclear grade, Bloom-Richardson histologic grade, estrogen receptor, progesterone receptor, HER2 overexpression, number of positive lymph nodes, and lymph node ratio >0.2. The distant metastasis-free survival and disease-free survival (DFS) rates were calculated using the Kaplan-Meier method. Univariate analysis with the log-rank test and multivariate analysis with the Cox proportional hazard method were used to identify prognostic factors for DFS. All statistical analyses were performed using R software version 3.2.2 (https://cran.r-project.org/).

## Results

### Locoregional recurrence

The median follow-up period was 76.5 months (range, 11–176 months). At the end of the study, 21 patients (7.3%) experienced LRR as the first failure, including 7 patients (4.1%) in the BCS+RT group and 14 patients (12.0%) in the TM group.

**Fig 1:**
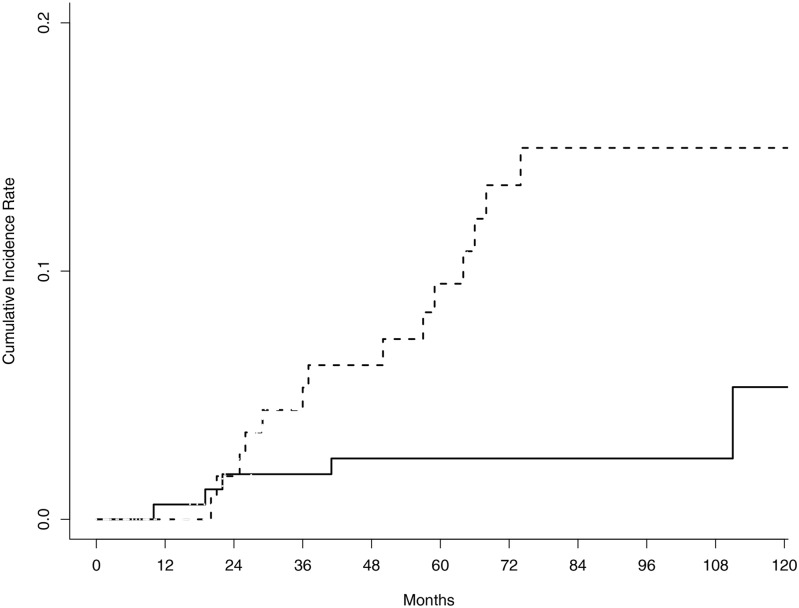
Comparison of the 5-year cumulative incidence rate of locoregional recurrence between the breast-conserving surgery plus radiotherapy (solid line) and total mastectomy alone groups (dotted line).

The 5-year cumulative incidence rate of LRR in the entire cohort was 5.4% (95% confidence interval [CI], 3.1–8.6%). The 5-year cumulative incidence rates of LRR were 2.5% (95% CI, 0.8–5.8%) in the BCS+RT group and 9.5% (95% CI, 4.8–16.1%) in the TM group (*p* = 0.016) (Fig 1). After competing risk regression analysis, histologic grade (relative risk [RR], 6.286; 95% CI, 2.137–18.49; *p* <0.001), HER2 overexpression (RR, 2.869; 95% CI, 1.027–8.02; *p* = 0.044) and TM (RR, 5.347; 95% CI, 1.767–16.18; *p* = 0.003) were associated with an increased risk of LRR (Table 2).

**Table 2:** Competing risk analysis for the cumulative incidence rate of locoregional recurrence.

	RR	95% CI	*p* value
Age ≥40 years	0.369	0.124–1.09	0.072
T stage	0.909	0.353–2.34	0.84
Close resection margin	1.444	0.432–4.83	0.55
Nuclear grade	3.886	0.96–15.74	0.057
Histologic grade	6.286	2.137–18.49	<0.001
Estrogen receptor	1.346	0.476–3.81	0.57
Progesterone receptor	0.654	0.313–1.37	0.26
HER2 overexpression	2.869	1.027–8.02	0.044
No. of positive LN	0.873	0.415–1.84	0.72
lymph node ratio	3.049	0.766–12.14	0.11
TM	5.347	1.767–16.18	0.003

RR, relative risk; CI, confidence interval; HER2, human epidermal growth factor receptor 2; LN, lymph node; TM, total mastectomy

### Survival rates

**Fig 2:**
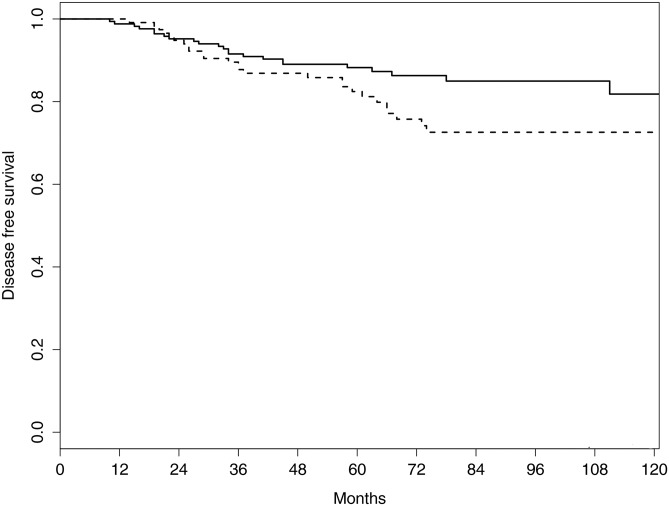
Comparison of the 5-year disease-free survival rate between the breast-conserving surgery plus radiotherapy (solid line) and total mastectomy groups (dotted line).

The 5-year distant metastasis-free survival rate was not significantly different between the groups (89.4% in the BCS+RT group vs. 89.1% in the TM group, *p* = 0.521). The 5-year DFS was 88.2% in the BCS+RT group versus 82.4% in the TM group (*p* = 0.092) (Fig 2). Age <40 years (*p* = 0.006), resection margin <2 mm (*p* = 0.031), a histologic grade of 3 (*p* <0.001), negative estrogen receptor (*p* = 0.024), and HER2 overexpression (*p* = 0.002) were associated with DFS in the univariate analysis. Negative progesterone receptor (*p* = 0.058) and lymph node ratio > 0.2 (*p* = 0.076) displayed borderline significance. T stage, number of positive lymph nodes, and nuclear grade were not associated with DFS (all *p* > 0.05). Multivariate analysis revealed that TM was associated with worse DFS compared with BCS+RT (hazard ratio, 2.024; 95% CI, 1.090–3.759; *p* = 0.026) (Table 3).

**Table 3:** Multivariate analysis for the disease-free survival rate.

	HR	95% CI	*p* value
Age ≥40 years	0.619	0.252–1.517	0.294
Resection margin <2 mm	2.082	0.958–4.524	0.064
Histologic grade 3	2.597	1.288–5.239	0.008
Positive estrogen receptor	0.768	0.359–1.642	0.496
Positive progesteron receptor	0.889	0.420–1.882	0.759
HER2 overexpression	1.422	0.766–2.639	0.265
lymph node ratio >0.2	1.934	0.960–3.897	0.065
TM	2.024	1.090–3.759	0.026

HR, hazard ratio; CI, confidence interval; HER2, human epidermal growth factor receptor 2; TM, total mastectomy

## Discussion

Currently, PMRT is indicated for high-risk breast cancer patients with ≥4 positive lymph nodes, tumor diameter > 5 cm, and/or involvement of the skin or fascia of skeletal muscle. However, the role of PMRT in patients with T1–2/N1 early-stage breast cancer has not been established.

Our study demonstrated that TM was associated with a significantly higher risk of LRR compared to BCS+RT among patients with T1–2/N1 breast cancer who received modern adjuvant systemic treatments. Furthermore, TM also produced worse DFS. This result might confirm the justifiability of PMRT in patients with T1–2/N1 breast cancer.

Our results were accordant with those of previous studies with similar designs [19–21]. Analyzing data from the Surveillance, Epidemiology, and End Results registry, Buchholz et al. reported that radiation use was independently associated with a survival benefit for patients with T1–2/N1 breast cancer compared with mastectomy alone [19]. Kim et al. reported that, in patients with T1–2/N1 breast cancer who received adjuvant adriamycin-based chemotherapy, BCS+RT significantly reduced both LRR and distant metastasis and appeared to provide better survival outcomes compared with TM [20]. An early meta-analysis clearly demonstrated that TM appeared to be inferior to BCS+RT for patients with early-stage breast cancer and positive lymph nodes [21]. However, PMRT had survival rates comparable to BCS+RT in patients with T1–2/N1 breast cancer.

Therefore, PMRT would produce better outcomes in patients with T1–2/N1 breast cancer compared with TM. Accumulating evidence demonstrated that PMRT reduced the risk of LRR in selected patients with T1–2/N1 breast cancer and several high-risk features compared with the outcomes of TM [7, 9–14]. However, definite risk factors have not been determined, and the criteria for identifying the high-risk group varied among the studies. For example, although young age was a common risk factor, the cutoff for young age varied among the studies [7–9, 13]. Moreover, in several studies including ours, age was not associated with an increased risk of LRR after mastectomy [11, 12, 14, 26]. In our study, HER2 overexpression was identified as an independent risk factor for LRR. However, Moo et al. reported that the molecular subtype based on immunohistochemical surrogate markers was not associated with LRR in patients with T1–2/N1 breast cancer treated with mastectomy [27].

Indeterminate risk factors raised the question whether PMRT should be used routinely or selectively for patients with T1–2/N1 breast cancer. As the latest Oxford overview demonstrated that PMRT significantly improved local control and survival rates after axillary clearance and systemic therapy in all patients with T1–2/N1 breast cancer [15], recent guidelines recommended routine use of PMRT in patients with node-positive breast cancer, irrespective of the number of positive lymph nodes [16, 17]. However, this meta-analysis included prospective randomized trials initiated prior to 2000 before the introduction of modern diagnostic procedures and systemic treatments. Concerning this limitation, several investigators disagreed with the routine use of PMRT because advances in diagnostic procedures and systemic therapy, as well as increases in the selective use of PMRT for high-risk patients, led to a lower LRR rate among patients who did not receive PMRT [13, 28]. Moo et al. reported that LRR incidence rate was similar between the PMRT and non-PMRT groups (3.2% vs. 4.3%) [13]. A study from MD Anderson Cancer Center also reported that patients treated in a more recent era (2000–2007) who did not receive PMRT exhibited an extremely low 5-year LRR rate of 2.8% [28]. The authors argued that detailed pathologic processing and serial sectioning of the sentinel lymph node biopsy increased the selective use of PMRT, and the introduction of more effective systemic regimens including taxanes and aromatase inhibitors resulted in favorable locoregional outcomes in patients who would not require PMRT.

Although the low rate of LRR from recent studies limited the role of PMRT for locoregional control, PMRT appeared to still have value in terms of DFS benefit in the era of modern diagnostic and therapeutic procedures. Chang et al. reported that, although advances in diagnostic procedures and systemic treatments reduced the LRR rate among patients who did not undergo PMRT to a level similar to that among patients who underwent PMRT, PMRT increased DFS significantly [26]. The present study also suggested a survival benefit of PMRT for patients with T1–2/N1 breast cancer.

Recently, the Medical Research Council and European Organization for Research and Treatment of Cancer initiated a prospective randomized trial, titled SUPREMO, to investigate the survival benefit of PMRT in early-stage breast cancer patients with intermediate risk [29]. The results of this trial will provide guidelines for PMRT in patients with T1–2/N1 breast cancer.

The present study had several limitations including a small number of patients and a retrospective nature. The decision concerning the selection of BCS+RT or TM was an important limitation. Patients with smaller tumors and fewer involved lymph nodes were more likely to receive BCS+RT. This bias might have seriously affected treatment outcomes. Despite our efforts to adjust for this bias via multivariate analysis, it is likely that other unknown biases influenced our results. Insufficient use of regional nodal irradiation was also a limitation of the present study. Recently, MA.20 investigators demonstrated a significant improvement in DFS for patients who received regional nodal irradiation encompassing all regional nodal areas [30]. The majority of patients included in the MA.20 trial had 1–3 positive lymph nodes and underwent BCS+RT. However, we performed regional nodal irradiation selectively, and the radiation field was confined to the supraclavicular area.

## Conclusions

In summary, our study suggested the potential requirement of PMRT in patients with T1–2/N1 breast cancer who received modern adjuvant systemic treatments. Even though modern adjuvant systemic treatments are known to improve treatment outcomes, the beneficial role of PMRT for T1–2/N1 breast cancer is likely to be valid in current clinical practice. Before the routine use of PMRT in patients with T1–2/N1 breast cancer, our results require confirmation by an ongoing prospective randomized trial.

## Supporting Information

S1 File Raw data excel file underlying the findings in present study. (CSV)
